# Efficacy of Sodium-Glucose Cotransporter 2 Inhibitors in Preventing Heart Failure in Patients Receiving Anthracycline-Based Cancer Therapy: A Systematic Review and Meta-Analysis

**DOI:** 10.7759/cureus.60086

**Published:** 2024-05-11

**Authors:** Godfrey Tabowei, Samuel K Dadzie, Prinka Perswani, Sheeza Nawaz, Mandeep Kaur, Merid Moqattash, Calvin R Wei, Shamsha Hirani

**Affiliations:** 1 Internal Medicine, California Institute of Behavioral Neurosciences & Psychology, Fairfield, USA; 2 Internal Medicine, Piedmont Athens Regional Medical Center, Athens, USA; 3 Internal Medicine, Liaquat University of Medical and Health Sciences, Hyderabad, PAK; 4 Medicine, Fatima Jinnah Medical University, Lahore, PAK; 5 Internal Medicine, Hospital Corporation of America (HCA) Capital Hospital, Tallahassee, USA; 6 Medicine, University of Pécs Medical School, Pécs, HUN; 7 Research and Development, Shing Huei Group, Taipei, TWN; 8 Cardiology, Baqai Hospital, Karachi, PAK

**Keywords:** all-cause mortality, systematic review and meta analysis, anthracycline, heart failure, sglt-2 inhibitor

## Abstract

Anthracyclines are effective chemotherapeutic agents widely used to treat various cancers, but their use is limited by the risk of cardiotoxicity and heart failure. While strategies like dose reduction have been explored, there are no well-established therapies to mitigate this risk. Emerging evidence suggests sodium-glucose cotransporter 2 inhibitors (SGLT2i) may have cardioprotective effects, providing a rationale for investigating their potential utility in anthracycline-treated patients. We conducted a systematic review and meta-analysis to synthesize available evidence on the efficacy of SGLT2i in reducing heart failure incidence and mortality in patients undergoing anthracycline-based cancer therapy. Relevant studies were identified through comprehensive database searches and screened based on predefined criteria. Data extraction and quality assessment were performed independently by two reviewers. Four observational studies, encompassing 5,590 patients, were included. The pooled analysis showed a higher but non-significant risk of developing heart failure in the non-SGLT2i group compared to the SGLT2i group (RR = 0.67, 95% CI: 0.40-1.41). The risk of all-cause mortality was significantly lower in patients receiving SGLT2i (RR = 0.55, 95% CI: 0.39-0.77). This meta-analysis suggests SGLT2i are associated with a lower risk of mortality and heart failure incidence in anthracycline-treated patients, although larger studies are needed to confirm these findings. The mechanisms underlying these potential benefits require further elucidation. Despite limitations, this analysis highlights the promising role of SGLT2i as a cardioprotective strategy in this high-risk population.

## Introduction and background

Anthracyclines, renowned for their efficacy in treating diverse solid tumors and hematological malignancies [1], are hindered by their potential to induce cardiac dysfunction, clinical heart failure, and arrhythmias [2]. Despite efforts like dose adjustment, formulation modification, and occasional use of chelation therapies such as dexrazoxane, no widely accepted treatments effectively mitigate anthracycline-induced cardiotoxicity and heart failure [3-4].

Research has indicated that sodium-glucose cotransporter 2 inhibitors (SGLT2i) exhibit promising cardioprotective properties by reducing inflammatory pathways [5], enhancing metabolic and endothelial functions [6], and decreasing the occurrence of atherosclerosis, arrhythmias, and major adverse cardiovascular events in individuals with diabetes [7-10]. Animal studies further support this notion, demonstrating that pretreatment with empagliflozin preserves cardiac function and reduces proinflammatory cytokines and cardiac fibrosis in anthracycline-associated cardiotoxicity [11-12].

Despite the well-established effectiveness of anthracyclines in cancer treatment, their use carries a notable risk of cardiotoxicity, including heart failure [2]. While various interventions have been explored to mitigate this risk, such as cardioprotective agents and monitoring strategies, the potential of SGLT2i remains underexplored. Given emerging evidence of their cardioprotective effects in other populations, there is a compelling rationale to investigate their utility specifically in anthracycline-treated patients. However, comprehensive comparative analyses assessing the efficacy of SGLT2i in this population are lacking in the current literature.

Through a systematic review and meta-analysis, we aim to address this knowledge gap and provide clinicians and researchers with a thorough synthesis of the available evidence. By synthesizing existing studies, our objective is to elucidate the potential of SGLT2i in reducing the incidence or severity of heart failure in patients undergoing anthracycline-based cancer therapy. This endeavor holds promise for informing clinical practice and advancing scientific understanding in this critical area.

## Review

Methodology 

Search Strategy 

A comprehensive search strategy was devised to identify studies related to the study's objective. Databases such as PubMed/MEDLINE, Embase, Cochrane Library, and Web of Science were searched systematically using a combination of keywords along with medical subject headings (MeSH) terms related to "SGLT2 inhibitors," "anthracyclines," "heart failure," and "cancer treatment." Efforts were undertaken to manually search the reference lists of eligible studies to ensure a comprehensive retrieval of relevant literature. The search strategy utilized MeSH terms as follows: (("Anthracyclines"[Mesh] OR anthracycline OR doxorubicin OR daunorubicin OR epirubicin OR idarubicin) AND ("Heart Failure"[Mesh] OR heart failure OR cardiac failure) AND ("SGLT2 Inhibitors"[Mesh] OR sodium-glucose cotransporter 2 inhibitors OR SGLT2i OR empagliflozin OR canagliflozin OR dapagliflozin)). This exhaustive search strategy aimed to minimize the risk of missing any pertinent studies and ensure the inclusivity of the review process. This systematic review and meta-analysis were synthesized and reported following the Preferred Reporting Items for Systematic Reviews and Meta-Analyses (PRISMA) guidelines. We did not put any restrictions on language or the year of publication.

Study Selection

Following the search, a meticulous screening process was undertaken to select studies meeting predefined inclusion and exclusion criteria based on the patient/population, intervention, comparison, and outcomes (PICO) framework. The population comprised adult patients who received anthracycline-based cancer therapy. The intervention included SGLT2i. The comparison group had a placebo or other therapies to prevent cardiovascular events. Outcomes included the incidence of heart failure, hospitalization due to heart failure, and all-cause mortality. We included observational studies as well as randomized controlled trials. Excluded were animal studies, reviews, case reports, and case series, as these were not original studies. This approach aligns with best practices in systematic reviews and meta-analyses, ensuring that our findings are based on the most applicable and reliable data available. Two independent reviewers assessed titles and abstracts for eligibility, followed by a full-text evaluation of potentially relevant articles. Any discrepancies in study selection were resolved through discussion or consultation with a third reviewer, ensuring the robust inclusion of appropriate studies.

Data Extraction and Quality Assessment

Subsequently, a standardized data extraction form was employed to systematically extract relevant information from the included studies. Two reviewers independently extracted data, encompassing author names, year of publication, sample size, participant demographics, and outcome measures. Consensus was reached on the extracted data through discussion or consultation with a third reviewer in the event of discrepancies. Methodological quality and risk of bias assessments were conducted for the included studies using established tools suitable for their respective study designs, such as the Cochrane Collaboration's tool for randomized controlled trials and the Newcastle-Ottawa Scale for cohort studies. Two reviewers independently evaluated the study quality, resolving any disagreements through discussion or by involving a third reviewer as necessary.

Statistical Analysis

The statistical analysis was performed using RevMan 5.4.1 software (The Cochrane Collaboration, London, UK). RevMan is a specialized software for systematic reviews and meta-analyses, offering tools for data management, statistical analysis, and graphical representation to facilitate evidence synthesis and interpretation. To quantify the effect of the intervention on the outcome variables, the risk ratio (RR) was calculated along with the corresponding 95% confidence interval (CI). A p-value less than 0.05 was considered statistically significant. The degree of heterogeneity among the study results was assessed using the I-square statistic. The I-square values of 50% or greater were indicative of substantial heterogeneity. In cases where significant heterogeneity was present, a random-effects model was employed to estimate the pooled effect. Conversely, when heterogeneity was not substantial, a fixed-effect model was utilized for the pooled effect estimation.

Results

**Figure 1:**
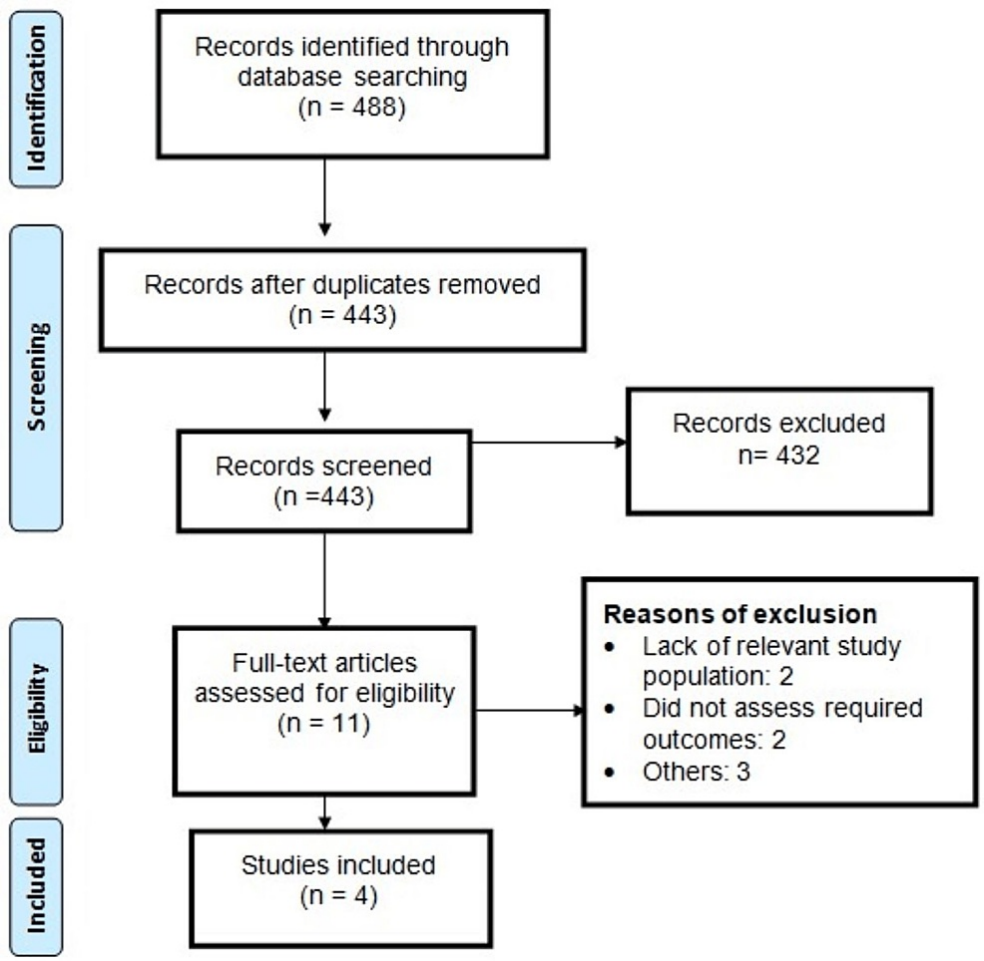
PRISMA flowchart of study selection PRISMA: Preferred Reporting Items for Systematic Reviews and Meta-Analyses

The initial online database search yielded 488 potentially relevant articles. After the initial screening process, 11 articles were deemed eligible for a more detailed evaluation based on the predefined inclusion and exclusion criteria. Ultimately, four studies met the criteria for inclusion in this meta-analysis, collectively encompassing a total of 5,590 patients. Figure 1 provides a visual representation of the study selection process. The characteristics of the included studies are summarized in Table 1. It is noteworthy that all of the studies included in the meta-analysis were observational in nature. Table 2 presents a quality assessment of the included studies. Out of the four studies included in this meta-analysis, three had high quality based on NCOS. 

**Table 1:** Characteristics of included studies SGLT2i: Sodium‑glucose cotransporter‑2 inhibitors

Study ID	Year	Study design	Region	Groups	Sample size	Age (years)	Males (n)
Abdel-Qadir et al. [13]	2023	Observational	Canada	SGLT2i	99	70	35
				Non-SGLT2i	834	71	318
Fath et al. [14]	2023	Observational	United States	SGLT2i	706	NR	NR
				Non-SGLT2i	706		
Gongora et al. [15]	2022	Observational	United States	SGLT2i	32	60	16
				Non-SGLT2i	96	60	36
Hwang et al. [16]	2023	Observational	Korea	SGLT2i	780	56	223
				Non-SGLT2i	2337	62	1263

**Table 2:** Quality assessment of included studies

Study ID	Selection	Comparison	Outcome/exposure assessment	Overall
Abdel-Qadir et al. [13]	4	2	2	Good
Fath et al. [14]	2	1	2	Fair
Gongora et al. [15]	3	2	2	Good
Hwang et al. [16]	4	2	3	Good

Incidence of Heart Failure

**Figure 2:**
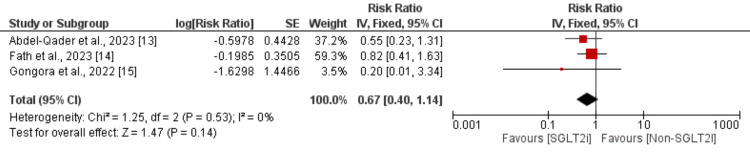
Effect of SGLT2i on the incidence of heart failure SGLT2i: Sodium‑glucose cotransporter‑2 inhibitors

Three studies were included in the pooled analysis to compare the risk of developing heart failure between the SGLT2i group and the non-SGLT2i group. The pooled analysis of these three studies showed a higher risk of developing heart failure in the non-SGLT2i group compared to the SGLT2i group, although the difference was not statistically significant (RR = 0.67, 95% CI: 0.40 to 1.41), as depicted in Figure 2. No significant heterogeneity was reported among the study results.

Heart Failure Hospitalization

**Figure 3:**
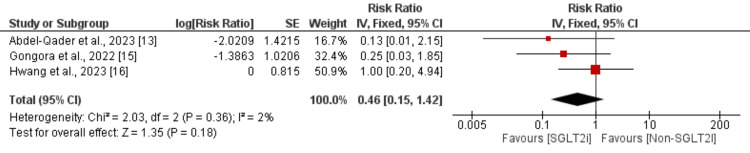
Effect of SGLT2i on risk of heart failure hospitalization SGLT2i: Sodium‑glucose cotransporter‑2 inhibitors

Three studies were included in the pooled analysis comparing the risk of developing heart failure between the two groups. The pooled analysis of three studies found that the heart failure risk is higher in the non-SGLT2i group than in the SGLT2i group. However, the difference between the two groups was not statistically significant (0.46, 95% CI: 0.15-1.42), as depicted in Figure 3. We did not find significant heterogeneity. 

Risk of All-Cause Mortality

**Figure 4:**
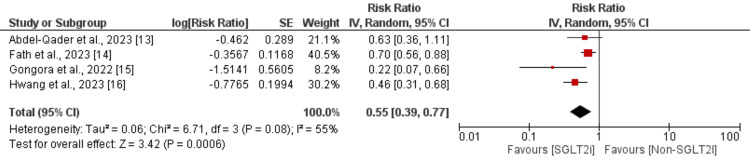
Effect of SGLT2i on the risk of mortality SGLT2i: Sodium‑glucose cotransporter‑2 inhibitors

We included all four studies that compared the all-cause mortality risk between SGLT2i and non-SGLT2i groups. Each of the incorporated studies demonstrated a potential advantage of SGLT2i with a reduced risk of all-cause mortality. However, three studies showed a significant difference between the two groups. The pooled RR showed that all-cause mortality risk was significantly lower in the SGLT2i group compared to the non-SGLT2i group (RR: 0.55, 95% CI: 0.39-0.77) (Figure 4). Significant heterogeneity was found among the study results. High heterogeneity is possibly due to variations in sample size and differences in population characteristics, including demographic variables and the region where the study was conducted.

Discussion

To the best of our knowledge, this is the first meta-analysis to demonstrate the benefits of SGLT2i in patients undergoing anthracycline-based cancer therapy. The findings of this study showed that SGLT2i are effective in reducing the risk of all-cause mortality. Additionally, the study demonstrated a lower risk of heart failure in patients receiving SGLT2i, although the difference was not statistically significant.

These findings contribute to the evolving field of cardio-oncology by providing evidence for a potential cardioprotective strategy in patients undergoing anthracycline-based chemotherapy, which is known to increase the risk of cardiotoxicity and heart failure. These results may influence treatment decisions by prompting the consideration of SGLT2i as adjunctive therapy in cancer patients at high risk for developing cardiotoxicity, particularly those with pre-existing cardiovascular risk factors or established cardiovascular disease.

The complete mechanism through which SGLT2i can alleviate heart failure in patients undergoing anthracycline therapy remains unclear. The study conducted by Quagliarliello et al. [17] exposed HL-1 adult cardiomyocytes to subclinical levels of trastuzumab and doxorubicin. They noted that simultaneous administration of dapagliflozin enhanced myocyte viability, as assessed through mitochondrial dehydrogenase activity, intracellular calcium homeostasis, and lipid peroxidation. Simultaneous administration of dapagliflozin also decreased the expression of inflammatory markers related to cardiotoxicity development in cardiomyocytes and suppressed the expression of signaling pathways associated with cardiomyocyte apoptosis [17]. However, it is important to note that these findings are from in vitro studies, and their translation to clinical settings may be limited due to differences in drug dosing, administration routes, and the complexity of the human physiological environment.

The study conducted by Giangiacomi et al. [18] aimed to assess the safety and efficacy of SGLT2i to treat cardiac dysfunction in patients receiving anthracycline. The study demonstrated the benefits of SGLT2i for anthracycline-related cardiac dysfunction. All patients included in this study exhibited notable enhancement in symptoms and echocardiographic assessment after including SGLT2i in their heart failure guideline-directed medical therapy. Additionally, the study reported that SGLT2i were safe and well-tolerated, with no cases of discontinuation. These clinical observations provide more direct evidence for the potential utility of SGLT2i in mitigating anthracycline-induced cardiotoxicity, although larger-scale clinical trials are needed to confirm these findings.

Several in vitro investigations [19-20] propose that inhibiting SGLT2 can impede tumor growth and trigger malignant cell death, offering a potential synergistic avenue in conjunction with other cancer treatments. Some of the suggested mechanisms [19-20] involve reducing glucose accessibility to metabolically active cancer cells by countering one of their adaptive strategies for proliferation, which involves upregulating sodium-glucose cotransporter expression to augment glucose intake [21]. While these preclinical studies provide insights into the potential anti-cancer effects of SGLT2i, caution should be exercised when extrapolating these findings to clinical settings due to differences in experimental conditions and the complexity of human cancer biology.

All studies included in this review reported a lower risk of mortality in individuals receiving SGLT2i. The lower risk of death in patients receiving SGLT2i is not only due to the lower risk of cardiovascular events in the SGLT2i group but also due to SGLT2i's anti-inflammatory action. The findings of this study also suggest that SGLT2i may reduce the growth of SGLT2-expressing cancer cells by impeding glucose uptake and blocking certain metabolic pathways. Moreover, SGLT2i decrease body weight via glycosuria, consequently enhancing insulin resistance [22-23].

Regarding the prevention of heart failure, all studies supported a lower incidence of heart failure in patients receiving SGLT2i, but none of the studies showed a significant difference between the two groups. A potential reason for the non-significant results could be the low sample size and inadequate statistical power to detect a significant difference. We recommend more trials to confirm the findings and guide clinical decision-making by professionals in choosing the best treatment options for this patient population.

These findings highlight the need for future clinical trials specifically designed to investigate the use of SGLT2i in cancer patients undergoing anthracycline-based therapy. Such trials could explore optimal dosing strategies, timing of SGLT2i initiation, and potential combination therapies with other cardioprotective agents. Additionally, mechanistic studies in clinical settings could provide further insights into the biological pathways through which SGLT2i exert their potential cardioprotective and anti-cancer effects, contributing to the development of more targeted and personalized treatment approaches.

Limitations

This meta-analysis has several important limitations that should be considered when interpreting the findings. First, our analysis included only four studies, all of which were observational in nature. While observational studies can provide valuable insights into real-world clinical settings, they are inherently limited by the potential for biases, such as selection bias and unmeasured confounding factors. These biases may have influenced the observed associations among SGLT2i, cardiac events, and mortality. Second, the generalizability of the findings to broader patient populations or clinical settings may be limited due to the heterogeneity in study designs, patient characteristics, and treatment protocols among the included studies. The studies varied in terms of the specific SGLT2i used, the timing of administration relative to anthracycline therapy, and the duration of follow-up, which could impact the observed effects on mortality and heart failure risk. Third, none of the included studies provided sufficient cause-specific mortality data, which precluded our ability to explore the potential underlying mechanisms responsible for the observed reduction in mortality associated with SGLT2i use.

Understanding the specific pathways and mechanisms through which SGLT2i may confer protective effects against heart failure and mortality in anthracycline-treated patients is crucial for informing clinical decision-making and guiding future research efforts. Moreover, the lack of patient-level data prevented us from performing subgroup analyses, which could have provided valuable insights into potential effect modifiers or patient characteristics that may influence the efficacy of SGLT2 inhibitors in this clinical context. Such analyses could help identify specific subpopulations that may benefit most from SGLT2i therapy or guide personalized treatment approaches.

Implications

This study contributes to filling existing knowledge gaps by systematically evaluating the efficacy of SGLT2i in reducing anthracycline-induced cardiotoxicity, a crucial area where comprehensive comparative analyses are lacking. By synthesizing available evidence, it addresses the scarcity of studies specifically assessing cardioprotective strategies in high-risk patient populations undergoing anthracycline-based cancer therapy, thus advancing understanding and potentially informing clinical practice in managing cardiotoxicity in this vulnerable patient group. The observed cardioprotective effects of SGLT2i in reducing anthracycline-induced heart failure could significantly impact patient management strategies. Selective prescription of SGLT2i based on cardiovascular risk assessment, coupled with closer monitoring of cardiac function and potential side effects, may become standard practice.

## Conclusions

This meta-analysis suggests that SGLT2i are associated with a decreased risk of all-cause mortality and heart failure in patients undergoing anthracycline-based cancer therapy, although larger studies are needed to confirm these findings. The mechanisms underlying these potential benefits remain unclear but may involve reduced inflammation, improved metabolic pathways, and decreased glucose availability for cancer cells. Despite limitations such as the small number of observational studies included, this analysis highlights the promising role of SGLT2i as a cardioprotective strategy in this high-risk patient population. Further research is warranted to guide clinical decision-making and optimize treatment approaches.
